# User Perceptions of Shared Sanitation among Rural Households in Indonesia and Bangladesh

**DOI:** 10.1371/journal.pone.0103886

**Published:** 2014-08-04

**Authors:** Kali B. Nelson, Jonathan Karver, Craig Kullman, Jay P. Graham

**Affiliations:** 1 Department of Epidemiology and Biostatistics, Milken Institute School of Public Health, The George Washington University, Washington, D.C., United States of America; 2 Washington, D.C., United States of America; 3 World Bank Water and Sanitation Program, Washington, D.C., United States of America; 4 Department of Environmental and Occupational Health and Department of Global Health, Milken Institute School of Public Health, The George Washington University, Washington, D.C., United States of America; Johns Hopkins Bloomberg School of Public Health, United States of America

## Abstract

**Background:**

The practice of sharing sanitation facilities does not meet the current World Health Organization/UNICEF definition for what is considered improved sanitation. Recommendations have been made to categorize shared sanitation as improved sanitation if security, user access, and other conditions can be assured, yet limited data exist on user preferences with respect to shared facilities.

**Objective:**

This study analyzed user perceptions of shared sanitation facilities in rural households in East Java, Indonesia, and Bangladesh.

**Methods:**

Cross-sectional studies of 2,087 households in East Java and 3,000 households in Bangladesh were conducted using questionnaires and observational methods. Relative risks were calculated to analyze associations between sanitation access and user perceptions of satisfaction, cleanliness, and safety.

**Results:**

In East Java, 82.4% of households with private improved sanitation facilities reported feeling satisfied with their place of defecation compared to 68.3% of households with shared improved facilities [RR 1.19, 95% CI 1.09, 1.31]. In Bangladesh, 87.7% of households with private improved facilities reported feeling satisfied compared to 74.5% of households with shared improved facilities [RR 1.15, 95% CI 1.10, 1.20]. In East Java, 79.5% of households who reported a clean latrine also reported feeling satisfied with their place of defecation; only 38.9% of households who reported a dirty latrine also reported feeling satisfied [RR 1.74, 95% CI 1.45, 2.08].

**Conclusion:**

Simple distinctions between improved and unimproved sanitation facilities tend to misrepresent the variability observed among households sharing sanitation facilities. Our results suggest that private improved sanitation is consistently preferred over any other sanitation option. An increased number of users appeared to negatively affect toilet cleanliness, and lower levels of cleanliness were associated with lower levels of satisfaction. However, when sanitation facilities were clean and shared by a limited number of households, users of shared facilities often reported feeling both satisfied and safe.

## Background

The Millennium Development Goals (MDG) Target 7C calls for halving the proportion of households without sustainable access to basic sanitation by 2015. A projected 2.4 billion people will still lack access to improved sanitation facilities in 2015, and global sanitation improvements towards Target 7C are estimated to fall short by half a billion people [1].

The goal of improved sanitation is to hygienically separate human excreta from human contact and therefore reduce exposure to fecal contamination [2]. UNICEF and the World Health Organization's Joint Monitoring Programme (JMP), the official body in charge of monitoring MDG development in the water and sanitation sector, define improved sanitation by the following types of facilities: toilets connected to sewers or septic systems, water-based toilets that flush into pits, simple pit latrines with slabs, and ventilated improved pit latrines [2]. To be considered improved, these facilities must be privately used by a single household: if any of these improved technologies is shared by more than one household, the household's sanitation access is considered unimproved. Unimproved facilities include any otherwise improved facility that is shared by more than one household as well as infrastructure that does not properly separate human excreta from potential human contact [3]. Unimproved sanitation facilities include, for example, the use of buckets, hanging latrines, or pit latrines without slab coverings. Engaging in open defecation is also considered unimproved sanitation.

### Shared Sanitation

Globally, increasing sanitation access has largely focused on shifting households away from engaging in open defecation or using unimproved facilities towards using private household facilities. In some areas, shared sanitation facilities may provide access to sanitation in communities where the installation of private latrines is not practical or sustainable due to issues of cost, space, or other barriers [4]. There is limited research on what specific factors influence households to share sanitation facilities in rural Asia. Research from other regions of the world, however, suggests that driving factors may include the lack of affordable sanitation options and low levels of awareness of the importance of sanitation [5]. Sharing sanitation facilities with extended family members and neighbors has been found to be more acceptable in certain cultures than in others, and shared sanitation may be a feasible alternative in rural and urban communities that lack access to improved sanitation, especially where open defecation persists [6], [7]. A recent study based on data from 51 countries found that shared sanitation appeared to be a risk factor for diarrhea, although the authors noted that social and economic context was an important factor for considering and judging shared sanitation [8].

The JMP acknowledges that shared sanitation responds to the need for increased sanitation access. Shared sanitation is being considered along with current proposals to define the Post-2015 MDG goals and indicators for sanitation, which recommend that improved sanitation be shared among no more than 5 households or 30 people, whichever is fewer. Historically, the JMP has hesitated to endorse shared sanitation in part because of potential decreased security and limited access to shared facilities [9]. Furthermore, the JMP has cited concerns about cleanliness, maintenance, lengthy distances from users' homes, long lines, cost barriers, and difficulty of use for elderly or disabled users and children [10]. Therefore, the JMP has recommended further research on the nature of shared facilities, including whether shared access can be tolerated by users [9].

Significant variability exists within the practice of shared sanitation, which suggests that diverse sanitation solutions may be more effective at meeting the needs of unique populations [11]. The location of the sanitation facility, who owns the facility, who is responsible for maintaining and cleaning the facility, and who uses the facility are examples of such divergence [12]. Further, because the division between improved and unimproved facilities is centered on technology instead of function, authorities may be dissuaded from incorporating context-specific solutions that are outside the realm of approved technologies, even if they properly address sanitation issues [13]. In a study of the limitations of the current JMP classification for shared sanitation, Mazeau and colleagues suggested new metrics for judging the adequacy of shared sanitation that would address the number of households that share a facility, the operations and maintenance of the facility, and the cleanliness of the facility [14]. Despite current proposals to include certain types of shared facilities in the JMP classification for improved sanitation, there is limited data on user satisfaction, especially among women and children, with respect to shared facilities in rural Asia.

### User Satisfaction with Sanitation

The literature on user satisfaction with shared sanitation is limited partially because little information is collected from users after the initial installation of sanitation infrastructure [15]. Measuring satisfaction is also limited because satisfaction is a complex concept that reflects personal and cultural experiences and expectations. Socio-cultural factors often drive utilization of sanitation facilities; therefore, the potential acceptability of new sanitation facilities must be modeled by socio-cultural acceptance of the technology as well as practical acceptance [12]. Satisfaction is integral to an individual's or community's decision to use available sanitation facilities.

### Sanitation in Indonesia and Bangladesh

Indonesia and Bangladesh highlight the tremendous, divergent gaps in access to sanitation and the global need for an intensified focus on addressing household barriers to promote access to improved sanitation. In 2009, half of all households in Indonesia and only one third of rural households had sustainable access to improved sanitation. Indonesia's MDG sanitation target strives for an overall increase to 62% coverage by 2015 [16]. In Bangladesh, almost two thirds of all households had sustainable access to improved sanitation in 2009. The MDG sanitation target for Bangladesh calls for an increase to 70% coverage by 2015 [17]. In 2010, approximately one third of the rural population of Indonesia and an estimated 7% of the rural population of Bangladesh engaged in open defecation [18].

Given the limited information that exists on user perceptions of shared sanitation in rural contexts, this study analyzes the relationship between different types of sanitation and user perceptions in rural areas of East Java, Indonesia, and Bangladesh. The specific aims of the study are to: 1) explore differences in user satisfaction among users who share toilet facilities compared to those who do not share and to those who engage in open defecation; 2) assess user satisfaction based on factors such as: a) facility cleanliness (East Java), b) ownership of the facility (Bangladesh), and c) perceptions of safety among women (East Java); and 3) characterize differences in households' plans to improve their sanitation facilities based on the type of sanitation facility used.

## Methods

### East Java

In December 2006, the World Bank's Water and Sanitation Program (WSP) began implementation of the Global Scaling Up Rural Sanitation project, a multi-year intervention focused on reducing diarrhea risk and increasing demand for sanitation in India, Indonesia, and Tanzania. In Indonesia, this project is locally known as Sanitasi Total dan Pemasaran Sanitasi (SToPs). In August and September 2008, the project's Global Impact Evaluation Team collected baseline data from households located in eight rural districts in East Java, Indonesia. Selection of the participants consisted of village selection followed by household selection. From the eight selected districts, 20 total villages (160 sub-villages) were included based on criteria such as water and sanitation access and poverty levels. The eight districts include: Probolinggo, Bondowoso, Situbondo, Banyuwangi, Ngawi, Madiun, Jombang, and Blitar. Thirteen households were randomly selected for inclusion in the baseline survey from a household list that included all households with children under the age of two years. Some sub-villages had too few households with children under the age of two; in these cases, households with children under the age of five were substituted. A total of 2,080 households were included in the baseline survey.

### Bangladesh

In 2003, the Government of Bangladesh launched a three-year national sanitation campaign focused on achieving total sanitation coverage and eradicating the practice of open defecation in rural populations. To assess the sustainability of sanitation interventions supported by the government and non-governmental organizations (NGO), WSP collected information on sanitation indicators and other household demographics for 3,000 households drawn from 50 of the Union Parishads (UP) that were declared open defecation free (ODF) in 2005. The Union Parishads represented the different geographic regions in Bangladesh and the different intervention approaches used to combat open defecation during the campaign. In places where a particular implementation approach was used more frequently, Union Parishads were selected randomly; however, in regions where a particular implementation approach was used less frequently, all UPs using this less popular approach were included to guarantee a broad representation of the approaches used in the sanitation campaign. Once the Union Parishad was selected, the research team randomly selected three villages or clusters of households with at least 100 households: one village located close to the UP government headquarters, one village located at an intermediate distance from UP headquarters, and a final village located a considerable distance away from UP headquarters (or one that was considered remote). Following this selection process, 100 households from each village or cluster of households were listed using a standard sampling format; from this list, 20 households were identified using a systematic random sampling method for a final sample size of 3,000 households.

### Variables

The variables of interest drawn from the East Java and Bangladesh household surveys were compiled from questions directed to the head of household. In East Java, a household includes everyone who lives in the house and shares meals together, including both family and non-family members. In Bangladesh, a household includes everyone who shares the household's food and who usually sleeps in the house at night. Individuals who visit periodically and contribute wages to the household are also included.

This research examines user satisfaction with the household sanitation facility. We recognize that household members may also use other sanitation facilities at work, school, neighbors' houses, and elsewhere. Nonetheless, we seek to understand users' preferences regarding the facilities they use at home given that the household is the basic unit of reference used in the MDG sanitation targets.

Differences exist in the questionnaires used by the two study sites, as noted below. Our study examined the following topics: sharing status; type of sanitation; level of satisfaction; perceived cleanliness (East Java only); improvement plans; open defecation (Bangladesh only); and ownership of the sanitation facility (Bangladesh only). Table S1 provides additional information on our research questions.

In East Java, the household questionnaire asked: “Overall, how satisfied are you with your main defecation facility?” Response options included: *very satisfied*, *somewhat satisfied*, *less than satisfied*, and *completely dissatisfied*. In the analysis for East Java, *very satisfied* and *somewhat satisfied* were combined; similarly, *less than satisfied* and *completely dissatisfied* answers were combined.

In Bangladesh, the household questionnaire asked: “How satisfied are you with your current place of defecation?” The answers included: *satisfied*, *moderately satisfied*, and *unsatisfied*. The answers *satisfied* and *moderately satisfied* were combined in the analysis for Bangladesh. Given the limited sample size in the different categorical responses, the binary satisfaction variables for East Java and Bangladesh provided more statistical power for the analyses.

For this study, we used the improved or unimproved designations based on the sanitation infrastructure only and did not use the facility's status as a private or shared facility to designate the toilet as improved or unimproved. Thus, if a facility met the structural standard for improved, not considering the private or shared status, then for the purposes of this study the facility was considered *improved private* or *improved shared*. We considered a facility to be private if used by only one household; we considered a facility to be shared if used by two or more households. Open defecation was considered a separate category and was not included in either the shared or unimproved categories. Variables S1 provides more information on variable definitions.

Potential confounders controlled for in the relevant analyses include: the age, gender, highest level of education and occupation of the head of household, the household's wealth category, religion, ethnicity (East Java only), ODF approach (Bangladesh only), and the status of the household's water source (improved or unimproved using JMP definitions). Definitions are provided in Covariates S1.

### Statistical Analysis

Pearson's chi-squared tests were conducted to test for independence among the covariates. A significant chi-square statistic suggests a dependent relationship between the tested variables. Relative risks (RRs) and 95% confidence intervals (CIs) were calculated using log-binomial regression. In cases where the model experienced problems of convergence, Poisson regression with robust error variance was used to calculate relative risks and 95% CIs. Relative risks were evaluated controlling for variables – selected *a priori* to the analysis – that were found to be significant in bivariate analyses. Relative risks can be interpreted as the likelihood of an outcome of interest occurring in one group of households compared to the likelihood of the same outcome occurring in a different group of households. Analyses were conducted using SAS 9.2 (Cary, NC).

### Ethical Consideration

The data used from Bangladesh and East Java are publicly available. The Bangladesh dataset was obtained from Craig Kullman, Water and Sanitation Program, World Bank. The East Java dataset was obtained from Bertha Briceno, Water and Sanitation Program, World Bank. Both datasets were anonymized before data analysis, and the research was determined to be exempt from human subject protection by The George Washington University Institutional Review Board given that it involved the analysis of pre-existing data that are publicly available. Further, ethical oversight was not obtained because the proposed uses and disclosures of protected health information involved no more than minimal risk to the privacy of individuals (45 CFR 46 164.512).

## Results

### East Java

Approximately 40% of surveyed households reported practicing open defecation. A further 11% used an unimproved sanitation facility and the remaining 49% of households used an improved facility. Among households with improved facilities, 71% of all households had a private improved facility and 29% shared an improved facility. Of households that shared improved or unimproved sanitation facilities, 51% of households shared the facility between two households, and 38% used a facility shared among three to five households. Only 10% shared the facility with six or more households. Approximately 68% of all surveyed households reported feeling satisfied with their sanitation facility. (Table 1).

**Table 1:** East Java Descriptive Statistics.

East Java Descriptive Statistics	
**Total number of households surveyed**	2,087
**Average size of household**	8.6 people
**Gender of head of household**	
*Male*	1,994 (95.5%)
*Female*	93 (4.5%)
**Age of head of household**	
*<25*	105 (5.0%)
*25 to 34*	739 (35.4%)
*35 to 44*	667 (32.0%)
*45 to 54*	340 (16.3%)
*55 to 64*	154 (7.4%)
*65+*	82 (3.9%)
**Ethnicity of household (n = 2,073)**	
*Javanese*	1,347 (65.0%)
*Maduranese*	681 (32.9%)
*Other (Osing, Chinese)*	45 (2.2%)
**Religion of household**	
*Islam*	2,059 (98.7%)
*Other (Protestant, Catholic, Hindu)*	28 (1.3%)
**Highest education level of head of household (n = 1,997)**	
*Primary or lower*	1,127 (56.4%)
*Secondary*	786 (39.4%)
*Tertiary or higher*	84 (4.2%)
**Primary occupation of head of household (n = 2,016)**	
*Agriculture, forestry, fishery, hunting, livestock*	1,008 (50%)
*Mining and exploration*	17 (0.8%)
*Manufacturing industry*	142 (7.0%)
*Electricity, gas and water*	6 (0.3%)
*Construction*	168 (8.3%)
*Trade, retail, restaurant and hotel*	293 (14.5%)
*Transportation, warehousing and communication*	99 (4.9%)
*Finance, insurance, building leasing, land and services*	14 (0.7%)
*Public service*	261 (12.9%)
*Other*	8 (0.4%)
**Household source of drinking water (rainy season) (n = 2,082)**	
*Improved*	*1,757 (84.4%)*
*Piped water, into home or yard*	130 (6.2%)
*Piped water, public tap, tube well, borehole*	482 (23.2%)
*Protected dug well*	757 (36.4%)
*Protected spring water*	388 (18.6%)
***Unimproved***	*325 (15.6%)*
*Unprotected dug well*	210 (10.1%)
*Unprotected spring water*	47 (2.3%)
*Other*	68 (3.3%)
**Household sanitation facility infrastructure and sharing status (n = 2,031)**	
*Improved, private*	723 (35.6%)
*Improved, shared*	262 (12.9%)
*Unimproved, private*	105 (5.2%)
*Unimproved, shared*	118 (5.8%)
*Open defecation*	823 (40.5%)
**Number of households sharing facility (n = 380)**	
*2 households*	194 (51.1%)
*3 households*	98 (25.8%)
*4 households*	26 (6.8%)
*5 households*	21 (5.5%)
*6 or more households*	38 (10.0%)
*Don't know*	3 (0.8%)
**User satisfaction with sanitation facility (n = 2,031)**	
*Very satisfied or somewhat satisfied*	*1,375 (67.7%)*
*Improved, private*	596 (29.3%)
*Improved, shared*	179 (8.8%)
*Unimproved, private*	44 (2.2%)
*Unimproved, shared*	63 (3.1%)
*Open defecation*	493 (24.3%)
***Less than satisfied or completely dissatisfied***	*656 (32.3%)*
*Improved, private*	127 (6.3%)
*Improved, shared*	83 (4.1%)
*Unimproved, private*	61 (3.0%)
*Unimproved, shared*	55 (2.7%)
*Open defecation*	330 (16.2%)
**Perceived cleanliness of sanitation facility by user (n = 1,208)**	
***Very clean or clean***	*1,011 (83.7%)*
*Improved, private*	665 (55.0%)
*Improved, shared*	222 (18.4%)
*Unimproved, private*	59 (4.9%)
*Unimproved, shared*	65 (5.4%)
***Dirty or very dirty***	*197 (16.3%)*
*Improved, private*	58 (4.8%)
*Improved, shared*	40 (3.3%)
*Unimproved, private*	46 (3.8%)
*Unimproved, shared*	53 (4.4%)

#### Sharing status, sanitation facility, and open defecation

We found that 82.4% of households with private improved latrines reported feeling satisfied with their place of defecation, while 70.2% of households who shared an improved facility between two households were satisfied [RR 1.19, 95% CI 1.06, 1.33] and only 68.3% of households with improved facilities shared between two or more households reported satisfaction [RR 1.19, 95% CI 1.09, 1.31]. In contrast, 48.0% of households with unimproved facilities reported feeling satisfied with their place of defecation, which is not significantly different from the 59.9% of households who engaged in open defecation who reported feeling satisfied [RR 1.10, 95% CI 0.94, 1.30]. (Table 2).

**Table 2:** Relative Risk for Households Reporting Satisfaction with their Main Defecation Facility (East Java).

*Analysis*		*Crude RR (95% CI)*	*p*	*Adjusted RR (95% CI)* *	*p*
**Sanitation facility**	Improved private facilities	1.38 (1.29, 1.47)	<0.0001	1.41 (1.32, 1.52)	<0.0001
	Improved shared facilities	1.14 (1.03, 1.26)	0.0097	1.25 (1.12, 1.40) Ω	<0.0001
	Unimproved facilities	1.25 (1.08, 1.45)	0.0032	1.10 (0.94, 1.30) Ω	0.2373
	Open defecation	1.00 (ref.)		1.00 (ref)	
**Private vs. shared (improved facilities)**	Improved private facility	1.21 (1.10, 1.32)	<0.0001	1.19 (1.09, 1.31)	0.0002
	Improved shared facility	1.00 (ref)		1.00 (ref)	
**Private vs. limited sharing**	Private facility	1.17 (1.05, 1.31)	0.0039	1.19 (1.06, 1.33)	0.0037
	Facility shared between only 2 households	1.00 (ref)		1.00 (ref)	
**Stratified limited sharing**	Facility shared between only 2 households	1.10 (0.95, 1.28)	0.2020	1.04 (0.89, 1.22) Ω	0.6379
	Facility shared among 3 or more households	1.00 (ref)		1.00 (ref)	
**Stratified limited sharing**	Facility shared between 2–3 households	1.14 (0.94, 1.40)	0.1844	1.08 (0.88, 1.32) Ω	0.4753
	Facility shared among 4 or more household	1.00 (ref)		1.00 (ref)	
**Facility cleanliness (as reported by household)** +	Clean	2.05 (1.71, 2.44)	<0.0001	1.74 (1.45, 2.08) Ω	<0.0001
	Dirty	1.00 (ref)		1.00 (ref)	

*All adjusted analyses were controlled for gender, age, ethnicity, religion, education level, and occupation of head of household, as well as household's income quartile and JMP status of drinking water source.

+ Households categorized as “open defecators” were excluded from this analysis.

Ω RRs and 95% CIs were calculated using Poisson regression with robust error variance.

#### Satisfaction levels and the number of households sharing the facility

In our analysis of satisfaction with place of defecation stratified by the number of households sharing the facility, the results were not significant. We found that 67.2% of households who shared their facility between two households reported feeling satisfied; 60.9% of households who shared their facility among three or more households reported feeling satisfied [RR 1.04, 95% CI 0.89, 1.22]. Satisfaction among households sharing between two or three households was reported at 66.0%; satisfaction among households who shared among four or more households was not significantly lower at 57.6% [RR 1.08, 95% CI 0.88, 1.32]. (Table 2). Finally, no significant differences in user satisfaction were observed in a comparison of three sharing levels: 66.0% of households who shared between two or three households were satisfied, 59.6% of households who shared among four or five households were satisfied, and 55.3% of households who shared among six or more households were satisfied [chi-square value 2.17, p  =  0.338].

#### Perceived cleanliness, sharing, and satisfaction

Households with clean sanitation facilities reported significantly different levels of satisfaction compared to households with dirty sanitation facilities. We found that 79.5% of households who perceived their sanitation facility to be clean also reported feeling satisfied with their place of defecation; only 38.9% of households who perceived their facility to be dirty reported feeling satisfied [RR 1.74, 95% CI 1.45, 2.08].

Households with private improved sanitation facilities did not perceive their places of defecation to be significantly cleaner than households that shared improved sanitation facilities between two households. Almost 92% of households with private improved latrines reported cleanliness; 91.4% of households who shared improved latrines between two households reported cleanliness [RR 1.00, 95% CI 0.82, 1.21]. Among households who shared an improved latrine, 91.4% of households who shared between two households perceived their facility to be clean; only 75.7% of households who shared among three or more households perceived their facility to be clean [RR 1.14, 95% CI 1.02, 1.27]. In a separate analysis of households who share an improved latrine, 88.7% of households who shared between 2 to 3 households perceived their facility to be clean; 68% of households who shared among 4 or more households perceived their facility to be clean [RR 1.21, 95% CI 1.00, 1.47]. (Table 3).

**Table 3:** Relative Risk for Households Reporting Clean Sanitation Facilities (East Java).

*Analysis*		*Crude RR (95% CI)*	*p*	*Adjusted RR (95% CI)* *	*p*
**Private vs. limited sharing (improved facilities)**	Improved private facility	1.01 (0.95, 1.06)	0.8144	1.00 (0.82, 1.21) Ω	0.9813
	Improved facility shared between only 2 households	1.00 (ref)		1.00 (ref)	
**Stratified limited sharing (improved facilities)**	Improved facility shared between only 2 households	1.21 (1.08, 1.36)	0.0015	1.14 (1.02, 1.27) Ω	0.0215
	Improved facility shared among 3 or more households	1.00 (ref)		1.00 (ref)	
**Stratified limited sharing (improved facilities)**	Improved facility shared between 2–3 households	1.30 (1.07, 1.59)	0.0080	1.21 (1.00, 1.47) Ω	0.0498
	Improved facility shared among 4 or more households	1.00 (ref)		1.00 (ref)	

*All adjusted analyses were controlled for gender, age, ethnicity, religion, education level, and occupation of head of household, as well as household's income quartile and JMP status of drinking water source.

Ω RRs and 95% CIs were calculated using Poisson regression with robust error variance.

#### Plans to improve sanitation facilities, sharing status, and satisfaction

We found that 42.6% of households with sanitation facilities shared between two or more households reported plans to upgrade their sanitation facility within the next year; in comparison, 44.2% of households with private improved facilities reported plans to upgrade their facility. This difference was not significant [RR 1.09, 95% CI 0.73, 1.63]. Approximately 47% of households with improved sanitation facilities shared between two households reported plans to upgrade their facility, which was not significantly more than the 44.2% of households with private improved facilities who reported plans to upgrade their sanitation facility [RR 1.14, 95% CI 0.74, 1.77]. In our analysis of households with improved facilities shared between two or more households, 46% of households who were satisfied with their place of defecation also reported plans to upgrade their facility within a year; 39.7% of households who were not satisfied with their place of defecation reported plans to upgrade their facility. No significant difference was observed [RR 0.97, 95% CI 0.63, 1.48]. (Table 4).

**Table 4:** Relative Risk for Households Reporting Plans to Improve Sanitation Facility or Build New Sanitation Facility (East Java).

*Analysis*		*Crude RR (95% CI)*	*p*	*Adjusted RR (95% CI)* *	*p*
**Private vs. shared (improved facilities)**	Improved shared facility	0.96 (0.65, 1.44)	0.8575	1.09 (0.73, 1.63)	0.6696
	Improved private facility	1.00 (ref)		1.00 (ref)	
**Private vs. limited sharing (improved facilities)**	Improved facility shared between only two households	1.07 (0.70, 1.65)	0.7531	1.14 (0.74, 1.77)	0.5448
	Improved private facility	1.00 (ref)		1.00 (ref)	
**Satisfaction level (improved shared facilities)**	Satisfied	1.16 (0.75, 1.80)	0.5056	0.97 (0.63, 1.48)	0.8729
	Unsatisfied	1.00 (ref)		1.00 (ref)	

*All adjusted analyses were controlled for gender, age, ethnicity, religion, education level, and occupation of head of household, as well as household's income quartile and JMP status of drinking water source.

#### Perceptions of female users

Additional questions were asked of female respondents regarding their perceptions of safety, privacy, and harassment. Almost 95% of women from households with private improved latrines reported that their facility is safe at night, which was not significantly different than the 91.2% of women from households with shared improved latrines that also reported that their facility is safe at night [RR 1.03, 95% CI 0.99, 1.08]. Approximately 89% of women from households who shared an improved latrine between two households reported that their facility is safe at night, which is significantly greater than the 77% of women from households who shared an improved latrine among three or more households who also reported that their facility is safe at night [RR 1.15, 95% CI 1.08, 1.23]. In a separate analysis, we found that 91.2% of women from households who shared an improved latrine with two or more households reported that their facility is safe at night; just 73.6% of women from households who openly defecate reported that their place of defecation is safe at night [RR 1.24, 95% CI 1.17, 1.31]. (Table 5). Additional results on perceptions of female users are provided in Table S2.

**Table 5:** Relative Risk for Women Reporting Feeling Safe at Night at Defecation Facility (East Java).

*Analysis*		*Crude RR (95% CI)*	*p*	*Adjusted RR (95% CI)* *	*p*
**Private vs. shared (improved facilities)**	Improved private facility	1.04 (1.00, 1.08)	0.06	1.03 (0.99, 1.08)	0.15
	Improved shared facility	1.00 (ref)		1.00 (ref)	
**Stratified limited sharing (improved facilities)**	Improved facility shared between only 2 households	1.16 (1.09, 1.23)	<0.0001	1.15 (1.08, 1.23)	<0.0001
	Improved facility shared among 3 or more households	1.00 (ref)		1.00 (ref)	
**Sanitation facility**	Improved shared facility	1.24 (1.17, 1.31)	<0.0001	–	
	Open defecation	1.00 (ref)		–	

*All adjusted analyses were controlled for gender, age, ethnicity, religion, education level, and occupation of head of household, as well as household's income quartile and JMP status of drinking water source.

### Bangladesh

Just over 2% of surveyed households reported engaging in open defecation, and only 8% used an unimproved facility. Over half of all households had a private improved facility (53%), and the remaining 37% of households had a shared improved facility. Among households that shared, 52% of the facilities were shared between two households, 45% of the facilities were shared with three to five households, and 3% of households shared with six or more households. Almost 83% of households with a private or shared improved facility reported feeling satisfied with their sanitation facility. Only 35% of households with a private or shared improved facility reported having plans to improve their latrine or build a new one within a year (Table 6).

**Table 6:** Bangladesh Descriptive Statistics.

Bangladesh Descriptive Statistics	
**Total number of households surveyed**	3,000
**Average size of household**	4.9 people
**Gender of head of household**	
*Male*	2,771 (92.4%)
*Female*	229 (7.6%)
**Age of head of household (years)**	
*<25*	77 (2.6%)
*25 to 34*	626 (20.9%)
*35 to 44*	782 (26.1%)
*45 to 54*	682 (22.7%)
*55 to 64*	490 (16.3%)
*65+*	343 (11.4%)
**Religion of household**	
*Islam*	2,553 (85.1%)
*Hinduism*	386 (12.9%)
*Buddhism*	61 (2.0%)
**Highest education level of head of household**	
*Primary or lower*	2,104 (70.1%)
*Secondary*	833 (27.8%)
*Tertiary or higher*	63 (2.1%)
**Primary occupation of head of household**	
*Agriculture, farming, poultry, fish, livestock*	1,027 (34.2%)
*Business*	184 (6.1%)
*Housewife*	151 (5.0%)
*Service*	232 (7.7%)
*Skilled labor*	194 (6.5%)
*Unskilled labor*	511 (17.0%)
*Small business*	393 (13.1%)
*Other*	308 (10.3%)
**Household source of drinking water**	
***Improved***	*2,930 (97.7%)*
*Piped water, into home or yard*	48 (1.6%)
*Public tap, standpipe*	19 (0.6%)
*Tube well, shallow or deep*	2,826 (94.2%)
*Protected well*	17 (0.6%)
*Protected spring*	20 (0.7%)
***Unimproved***	*70 (2.3%)*
*Unprotected well*	3 (0.1%)
*Unprotected spring*	21 (0.7%)
*PSF (Pond Sand Filter - surface water)*	46 (1.5%)
**Household sanitation facility, sharing status, and ownership status**	
***Improved facility, private***	*1,588 (52.9%)*
*Own*	1570 (52.3%)
*Jointly owned*	0 (0%)
*Live on rent*	18 (0.6%)
***Improved facility, shared***	*1,098 (36.6%)*
*Own*	225 (7.5%)
*Jointly owned*	692 (23.1%)
*Owned by others/neighbor*	181 (6.0%)
***Unimproved facility***	*237 (7.9%)*
***Open defecation***	*77 (2.6%)*
**Number of households sharing sanitation facility (n = 515)**	
*2 households*	268 (52.0%)
*3 households*	145 (28.2%)
*4 households*	64 (12.4%)
*5 households*	24 (4.7%)
*6 or more households*	14 (2.7%)
**User satisfaction with sanitation facility (n = 2,487)**	
***Satisfied or moderately satisfied***	*2,060 (82.8%)*
*Improved, private*	1,377 (55.4%)
*Improved, shared*	683 (27.5%)
***Unsatisfied***	*427 (17.2%)*
*Improved, private*	193 (7.8%)
*Improved, shared*	234 (9.4%)
**Household plans to improve the present latrine or build a new one within twelve months (n = 2,466)**	
***Yes***	*871 (35.3%)*
*Improved, private*	516 (20.9%)
*Improved, shared*	355 (14.4%)
***No***	*1,595 (64.7%)*
*Improved, private*	1,044 (42.3%)
*Improved, shared*	551 (22.3%)
**Member of household defecates in the open other than in flood time (n = 2,680)**	
***Yes***	*176 (6.6%)*
*Improved, private*	62 (2.3%)
*Improved, shared*	114 (4.3%)
***No***	*2,504 (93.4%)*
*Improved, private*	1,521 (56.8%)
*Improved, shared*	983 (36.7%)

#### Sharing status and sanitation facility

Among households with private improved facilities, 87.7% of households reported feeling satisfied. In comparison, only 74.5% of households with improved facilities shared by two or more households were satisfied [RR 1.15, 95% CI 1.10, 1.20]. Approximately 74% of households with improved facilities who shared between two households reported feeling satisfied with their place of defecation compared to only 64.8% of households who shared an improved facility among three or more households [RR 1.15, 95% CI 1.03, 1.29]. (Table 7).

**Table 7:** Relative Risk for Households Reporting Satisfaction with their Current Place of Defecation (Bangladesh).

*Analysis*		*Crude RR (95% CI)*	*p*	*Adjusted RR (95% CI)* *	*p*
**Private vs. shared (improved facilities)**	Improved private facility	1.18 (1.13, 1.23)	<0.0001	1.15 (1.10, 1.20) Ω	<0.0001
	Improved shared facility	1.00 (ref)		1.00 (ref)	
**Private vs. limited sharing (improved facilities)**	Improved private facility	1.18 (1.10, 1.27)	<0.0001	1.15 (1.07, 1.24) Ω	0.0001
	Improved facility shared between 2 households	1.00 (ref)		1.00 (ref)	
**Stratified limited sharing (improved facilities)**	Improved facility shared between 2 households	1.15 (1.02, 1.29)	0.0209	1.15 (1.03, 1.29) Ω	0.0122
	Improved facility shared among 3 or more households	1.00 (ref)		1.00 (ref)	
**Stratified limited sharing (improved facilities)**	Improved facility shared between 2–3 households	1.14 (0.97, 1.34)	0.1157	1.15 (0.98, 1.34) Ω	0.0910
	Improved facility shared among 4 or more households	1.00 (ref)		1.00 (ref)	
**Facility ownership (improved shared facilities)**	Privately own sanitation facility	1.10 (1.02, 1.19)	0.0184	1.10 (1.01, 1.19) Ω	0.0251
	Jointly own sanitation facility	1.00 (ref)		1.00 (ref)	

* Controlling for head of household's gender, age, religion, education level, and occupation, household income quintile, approach to ODF, and JMP status of drinking water source.

Ω RRs and 95% CIs were calculated using Poisson regression with robust error variance.

#### Satisfaction levels and the number of households sharing the facility

We found that 71.4% of households sharing an improved facility between two to three households felt satisfied, which was not significantly different than the 62.7% of households sharing an improved facility among four or more households who also felt satisfied with their facility [1.15, 95% CI 0.98, 1.34]. No significant differences in user satisfaction were seen in a separate analysis that assessed three levels of sharing: households who shared an improved facility between two or three households, households who shared an improved facility among four or five households, and households who shared an improved facility among six or more households [chi-square value: 5.96, p  =  0.051]. (Table 7).

#### Ownership of the sanitation facility

Significant differences in user satisfaction were observed between households who independently owned a shared latrine compared to households who shared ownership of the shared latrine with other households. Exactly 80% of households who privately owned an improved shared latrine felt satisfied with their place of defecation; 72.7% of households who jointly owned an improved shared latrine felt satisfied [RR 1.10, 95% 1.01, 1.19]. (Table 7).

#### Plans to improve sanitation facilities, sharing status, and satisfaction

Among households with shared improved facilities, 39.2% reported plans to improve their current latrine or build a new latrine within a short period of time. Among households with private improved facilities, 33.1% reported similar improvement plans [RR 1.14, 95% CI 1.02, 1.27]. In contrast, in an analysis of household plans to improve a current latrine or build a new latrine, 38.4% of households sharing an improved latrine between two households reported plans to improve their latrine; 33.1% of households with a private improved latrine reported improvement plans [RR 1.14, 95% CI 0.96, 1.35]. Among households with an improved shared facility, 63.4% of households who reported feeling unsatisfied with their facility also reported plans to improve their current latrine. In contrast, only 31.1% of households with improved shared facilities who reported feeling satisfied with their facility also reported plans to improve their latrine [RR 2.04, 95% CI 1.76, 2.37]. (Table 8).

**Table 8:** Relative Risk for Households Reporting Plans to Improve Sanitation Facility or Build New Sanitation Facility (Bangladesh).

*Analysis*		*Crude RR (95% CI)*	*p*	*Adjusted RR (95% CI)* *	*p*
**Private vs. shared (improved facilities)**	Improved shared facility	1.18 (1.06, 1.32)	0.0020	1.14 (1.02, 1.27)	0.0248
	Improved private facility	1.00 (ref)		1.00 (ref)	
**Limited sharing vs. private (improved facilities)**	Improved facility shared between 2 households	1.16 (0.98, 1.37)	0.0825	1.14 (0.96, 1.35)	0.1471
	Improved private facility	1.00 (ref)		1.00 (ref)	
**Satisfaction level (improved shared facilities)**	Unsatisfied	2.04 (1.76, 2.37)	<0.0001	–	–
	Satisfied	1.00 (ref)		1.00 (ref)	

* Controlling for head of household's gender, age, religion, education level, and occupation, household income quintile, approach to ODF, and JMP status of drinking water source.

#### Open defecation

Almost 18% of households who shared an improved sanitation facility and who also felt unsatisfied with their sanitation facility continued to openly defecate; in contrast, only 6.3% of households who were satisfied reported continued open defecation [RR 2.68, 95% CI 1.81, 3.96]. In a similar analysis, 11.6% of households that shared their improved facility between two households continued to openly defecate; only 3.9% of households with private improved latrines also reported open defecation [RR 2.18, 95% CI 1.38, 3,45]. (Table 9).

**Table 9:** Relative Risk for Households Reporting Open Defecation Plans (Bangladesh).

Analysis		Crude RR (95% CI)	p	Adjusted RR (95% CI)*	p
**Satisfaction level (improved shared facilities)**	Unsatisfied	2.85 (1.91, 4.25)	<0.0001	2.68 (1.81, 3.96)	<0.0001
	Satisfied	1.00 (ref)		1.00 (ref)	
**Private vs. limited sharing (improved facilities)**	Improved facilities shared between only 2 households	2.95 (1.96, 4.46)	<0.0001	2.18 (1.38, 3.45) Ω	0.0008
	Improved private facilities	1.00 (ref)		1.00 (ref)	

* Controlling for head of household's gender, age, religion, education level, and occupation, household income quintile, approach to ODF, and JMP status of drinking water source.

Ω RRs and 95% CIs were calculated using Poisson regression with robust error variance.

## Discussion

### East Java

#### Sharing status, sanitation facility, and open defecation

Our results suggest that households with private improved facilities were more satisfied than households that shared improved facilities, even when sharing the facility was limited to sharing between only two households. Households were much more likely to be satisfied with a private improved facility than were households practicing open defecation. Similarly, households sharing an improved facility were more likely to be satisfied than households practicing open defecation. No significant difference in satisfaction was found between households with unimproved facilities and households practicing open defecation. These results suggest that sharing improved sanitation facilities is preferred over the practice of open defecation and that private improved sanitation is consistently preferred over any other sanitation option.

#### Satisfaction levels and the number of households sharing the facility

Among sharers, the number of households sharing did not seem to significantly alter satisfaction levels, although we were not able to look at all combinations of the number of households sharing a given facility in our analysis. No significant difference was observed in satisfaction in users of facilities shared between two households compared to users of facilities shared by three or more households. This finding is contrary to our results found in Bangladesh. Other research has indicated that a greater number of households sharing a sanitation facility lead to lower levels of satisfaction. For example, in a study on user satisfaction with sanitation facilities in Kampala, Uganda, the main determinant for reporting dissatisfaction was sharing a facility with too many users [7].

#### Perceived cleanliness

As mentioned previously, the JMP acknowledges that shared facilities potentially could be regarded as improved facilities but notes concerns about cleanliness and hygiene [10]. User perceptions of latrine cleanliness were not significantly different in users of private latrines compared to users of facilities shared between two households. Our research suggests that sharing sanitation facilities between a limited number of households, namely two, may not alter perceived cleanliness. Similarly, cleanliness was seen as a driver for latrine adoption in a separate study in Benin, West Africa [19]. In Bhopal, India, an assessment of seven communal toilet facilities revealed that 65% of users were satisfied with the condition of the communal toilets, and the facilities were liked mostly because of their convenience and privacy; they were disliked primarily due to their lack of cleanliness and smell [20]. In a study in Kampala, Uganda, the second most frequent reason for reporting dissatisfaction with sanitation facilities was that facilities were not clean and smelled bad [7]. Perceived cleanliness appears to play an important role in satisfaction with sanitation facilities in East Java; similar research in other regions corroborates these results.

#### Plans to improve sanitation facilities

Household plans to improve a current latrine or build a new latrine within a short period of time were not affected by sharing status or level of user satisfaction. These results indicate that other barriers to procuring a private sanitation facility may exist or that private sanitation is not a main priority relative to other household needs.

#### Perceptions of female users

In our analysis of surveyed communities in East Java, shared improved facilities were perceived to be safe for use by women during the evening hours. No significant differences were observed in perceived nighttime safety among women from households with private improved latrines compared to women from households with shared improved latrines. However, among sharers of improved latrines, women from households who shared between two households were more likely to feel safe than women from households who shared among three or more households. Further, women from households with shared improved facilities were significantly more likely to report feeling safe compared to women from households that openly defecated. These results suggest that efforts made to reduce the number of households that share sanitation facilities could reduce levels of fear among female users. Other research in rural Benin has shown that increasing privacy and safety, especially for women, were documented reasons for wanting a latrine [19]. Likewise, a safe and private sanitation facility was more significant for women in urban Ghana and urban Uganda than for their male counterparts [14]. While more research is needed on the perceptions of women and children to better understand the factors that contribute to safety in different sanitation contexts, safety remains a key matter in the assessment of sanitation facilities.

### Bangladesh

#### Sharing status and sanitation facility

Our results suggest that households were more satisfied with private improved facilities than with shared improved facilities, even if only two households shared the facility. In contrast to East Java, the results in Bangladesh indicate that satisfaction was significantly more likely among households sharing an improved facility between two households compared to households sharing an improved facility among three or more households.

#### Satisfaction levels and the number of households sharing the facility

Satisfaction was not significantly different between households with improved facilities shared between two to three households compared to households with improved facilities shared among four or more households. These results are similar to our results from East Java. It should be noted that the Government of Bangladesh considers hygienic sanitation facilities shared between two households to be improved sanitation [21]. It is unclear, however, how and why this decision by the government was made and whether it was based on users' perceptions, feasibility issues, or other driving factors.

#### Ownership of the sanitation facility

Satisfaction was found to be significantly greater among households who privately owned a shared facility compared with those who jointly owned a shared facility. Ownership of a housing structure with a sanitation facility may provide greater satisfaction, as landlords can potentially charge more for housing. In rural Benin, researchers found that households often wanted a latrine because they were embarrassed to ask visitors to openly defecate. Households wanted to gain the respect of visitors, and latrine ownership was seen as a way to raise a household's status among their neighbors [19].

#### Plans to improve sanitation facilities

Households sharing an improved facility between two or more households were more likely to report plans to improve their current latrine or to build a new latrine compared to households who did not share; however, households who shared an improved latrine between two or more households were not significantly more likely to have these plans compared to households with improved private facilities. These results may indicate that households with private improved latrines do not differ in their plans to improve their current latrine or to build a new latrine when compared to households sharing an improved latrine between two households. Further, unsatisfied households were more likely to report improvement plans than were satisfied households. These results suggest that households who share their sanitation facilities with others and who are unsatisfied with their current facility would like to see an improvement in their existing sanitation facility.

#### Open defecation

The practice of open defecation is wrought with difficulties, discomforts, and dangers, and in many cases, households would like to improve their sanitation situation [19]. It should be noted, however, that households with better sanitation options may still resort to open defecation. In Bangladesh, households with shared improved facilities who reported dissatisfaction with their sanitation facility were more likely to report practicing open defecation compared to households with shared improved facilities who reported satisfaction with their facility. These results suggest that user satisfaction may play a role in helping move households “up” the sanitation ladder and away from the practice of open defecation. Sharing status and sanitation infrastructure are two important factors that must be considered when analyzing sanitation options. Understanding the factors that affect a latrine user's level of satisfaction must be included in planning strategies.

Studies in urban settings have reported that shared sanitation may serve to reduce open defecation in communities where it continues to persist. For example, community sanitation systems were installed in urban Bhopal, India, because they were considered the best option for quickly addressing the sanitation needs of the urban poor. Upon evaluation of the community sanitation systems, the significant determinants of communal latrine use by members of households without a latrine included distance to the latrine, opening hours, facility age, cleanliness, and cost. These shared sanitation facilities were found to reduce open defecation but not eliminate it completely. Interestingly, twice as many males as females used the communal latrines [22]. A study in urban Bangladesh found that public sanitation facilities increased weight-for-height scores in children. The authors hypothesized that the improvements were a result of reductions in environmental contamination by children's feces [23]. Reducing open defecation remains integral to overall improvement in the sanitation sector, and moving people to shared sanitation, under certain conditions, may be beneficial [24].

### Limitations

This cross-sectional study provides insight into factors that affect satisfaction and draws inferences about different exposures. Alternative explanations for the results may exist and should be considered. Many of the findings in East Java, Indonesia, and Bangladesh were analogous. However, the variability in our results suggests that the findings may be limited to rural East Java and rural Bangladesh. These results may not be applicable to urban and peri-urban populations within the countries studied or to other countries. Furthermore, this study does not analyze the effects of broader factors such as policies, economic factors, or macro-social influences such as ethnicity or religion on latrine access and use. In certain cases, the limited sample size within different categories of the number of households sharing a sanitation facility hindered the ability to statistically test satisfaction levels.

Additionally, efforts are needed to expand the approaches used to measure satisfaction in different cultural contexts. For example, researchers in India have developed the Pachod Paisa scale, a metric created with the local culture in mind. The numeric Pachod Paisa scale, so named for the one hundred “paisa” that make up the Indian rupee, has been used for measuring patient satisfaction with health care facilities and other related variables concerning personal impressions. The scale was also used in a sanitation study in rural Maharashtra, India, to measure the factors driving latrine use. The Pachod Paisa scale offers insight into satisfaction measurements on the Indian subcontinent and illustrates a workable, culturally appropriate alternative to other scales [25].

## Conclusions

From the perspective of users, private sanitation facilities appear to be the preferred form of sanitation. In both rural and urban communities where private sanitation may not be a realistic option, shared sanitation facilities that maintain an acceptable level of cleanliness and limit the numbers of users are feasible alternatives. User satisfaction is one crucial piece in the sanitation sector that may inhibit or encourage acceptance of and commitment to a change or “step up” the sanitation ladder. More research is needed to understand household satisfaction, and future research should integrate metrics for measuring satisfaction into surveys. As seen in rural Bangladesh, when moving communities away from open defecation, satisfaction with the sanitation facility is crucial in order to prevent open defecation practices from recurring. In addition, perceived cleanliness of the facility was positively associated with household satisfaction in East Java. Simple distinctions between improved and unimproved technologies tend to oversimplify the spectrum of sanitation facilities and misrepresent the variability found within the shared sanitation sector. Shared sanitation facilities that are well maintained, safe for women, and shared by a limited number of households may be an acceptable form of sanitation. Efforts to improve sanitation should aim to incorporate these findings in order to more robustly track progress.

## Supporting Information

Table S1 **Research questions.**
(DOCX)

Table S2 **Female perceptions of household sanitation facilities in East Java.**
(DOCX)

Variables S1 **Variable definitions.**
(DOCX)

Covariates S1 **Covariate definitions for East Java and Bangladesh.**
(DOCX)

## References

[1] WHO/UNICEF (2013) Progress on Drinking Water and Sanitation: 2013 Update. Available: http://www.unicef.org/wash/files/JMP2013final_en.pdf. Accessed 15 September 2013.

[2] WHO/UNICEF (2012) Proposal for consolidated drinking water, sanitation and hygiene targets, indicators and definitions. Available: http://www.wssinfo.org/fileadmin/user_upload/resources/A-proposal-for-consolidated-WASH-goal-targets-definitions-and-indicators_version7_Nov22_final.pdf. Accessed 05 July 2013.

[3] WHO/UNICEF (2010) Improved and unimproved water and sanitation facilities. Available: http://www.wssinfo.org/definitions-methods/watsan-categories/. Accessed 07 October 2012.

[4] Norman G (February 2011) When are communal or public toilets an appropriate option? Water and Sanitation for the Urban Poor. Available: http://www.wsscc.org/sites/default/files/publications/wsup_topicbrieficommunalpublictoilets_2011.pdf. Accessed 07 October 2012.

[5] Rodgers AF, Ajono LA, Gyapong JO, Hagan M, Emerson PM (2007) Characteristics of latrine promotion participants and non-participants; inspection of latrines; and perceptions of household latrines in Northern Ghana. Trop Med Int Health 12: 772–782 10.1111/j.13653156.2007.01848.x 17550475

[6] Günther I, Niwagaba BC, Lüthi C, Horst A, Mosler H, et al. (December 2012) When is shared sanitation improved sanitation? Available: http://www.eawag.ch/forschung/ess/gruppen/ehpsy/publications/pdfs/Policy_Brief_2_2012.pdf. Accessed 05 July 2013.

[7] Tumwebaze IK, Orach CG, Niwagaba C, Lüthi C, Mosler HJ (2012) Sanitation facilities in Kampala slums, Uganda: users' satisfaction and determinant factors. Int J Environ Health Res 23: 191–204 10.1080/09603123.2012.713095 22873693

[8] Fuller JA, Clasen T, Heijnen M, Eisenberg JNS (2014) Shared Sanitation and the Prevalence of Diarrhea in Young Children: Evidence from 51 Countries, 2001–2011. Am J Trop Med Hyg 91: 173–80 10.4269/ajtmh.13-0503 24865679 PMC4080558

[9] WHO/UNICEF (2008) Progress on Drinking Water and Sanitation: Special Focus on Sanitation. Available: http://www.wssinfo.org/fileadmin/user_upload/resources/1251794333-JMP_08_en.pdf. Accessed: 10 October 2012.

[10] WHO/UNICEF (2010) JMP Technical Task Force Meeting on Sanitation and Methods for Estimating Progress. Available: http://www.wssinfo.org/fileadmin/user_upload/resources/JMP-Sanitation-Method-Task-Force-Meeting-Report-July-2010-final.pdf. Accessed: 22 February 2013.

[11] Mazeau A, Reed B, Sansom K, Scott R (2013) Emerging categories of urban shared sanitation. Water Environ J 10.1111/wej.12075

[12] Mazeau AP, Reed B (2010) Assessing people's views of infrastructure: methodologies to study urban shared sanitation. Available: http://hal.archives-ouvertes.fr/docs/00/52/14/09/PDF/9-WWW-YES-Mazeau-Paper-2010-04-08.pdf. Accessed 22 February 2013.

[13] Kvarnström E, McConville J, Bracken P, Johansson M, Fogde M (2011) The sanitation ladder - a need for a revamp? J Water Sanit Hyg Dev 1: 3–12 10.2166/washdev.2011.014

[14] Mazeau A, Tumwebaze IK, Lüthi C, Sansom K (2013) Inclusion of shared sanitation in urban sanitation coverage? Evidence from Ghana and Uganda. Waterlines 32: 334–348 10.3362/1756-3488.2013.034

[15] Roma E, Buckley C, Jefferson B, Jeffrey P (2010) Assessing users' experience of shared sanitation facilities: A case study of community ablution blocks in Durban, South Africa. Water SA 36: 589–594 10.4314/wsa.v36i5.61992

[16] Ministry of National Development Planning/National Development Planning Agency (BAPPENAS) (2010). Report on the Achievement of the Millennium Development Goals Indonesia 2010. Available: http://planipolis.iiep.unesco.org/upload/Indonesia/Indonesia_MDG_2010.pdf. Accessed 05 July 2013.

[17] General Economics Division, Planning Commission, Government of the People's Republic of Bangladesh (GED) (February 2012) The Millennium Development Goals: Bangladesh Progress Report 2011. Available: http://www.un-bd.org/pub/MDG%20Progress%20Report%202011.pdf. Accessed 05 July 2013.

[18] WHO/UNICEF (April 2014) Estimates on the use of water sources and sanitation facilities: Bangladesh. Available: http://www.wssinfo.org/documents/?tx_displaycontroller%5Btype%5D=country_files. Accessed 12 July 2014.

[19] Jenkins MW, Curtis V (2005) Achieving the ‘good life’: why some people want latrines in rural Benin. Soc Sci Med 61: 2446–59 10.1016/j.socscimed.2005.04.036 15949883

[20] de Souza K (April 2010) Communal Toilets in Urban Poverty Pockets: Use and user satisfaction associated with seven communal toilet facilities in Bhopal, India. WaterAid. Available: http://www.wateraid.org/~/media/Publications/communal-toilets-user-satisfaction-bhopal-india-briefing-note.pdf. Accessed: 10 October 2012.

[21] Zheng Y, Hakim SAI, Nahar Q, Agthoven Av, Flanagan SV (2013) Sanitation coverage in Bangladesh since the millennium: consistency matters. J Water Sanit Hyg Dev 3: 240–251 10.2166/washdev.2013.154

[22] Biran A, Jenkins MW, Dabrase P, Bhagwat I (2011) Patterns and determinants of communal latrine usage in urban poverty pockets in Bhopal, India. Trop Med Int Health 16: 854–862 10.1111/j.1365-3156.2011.02764.x 21414114

[23] Buttenheim AM (2008) The sanitation environment in urban slums: implications for child health. Popul Environ 30: 26–47 10.1007/s11111-008-0074-9 25825551 PMC4377083

[24] Montgomery MA, Desai MM, Elimelech M (2010) Comparing the effectiveness of shared versus private latrines in preventing trachoma in rural Tanzania. Am J Trop Med Hyg 82: 693–695 10.4269/ajtmh.2010.09-0540 20348521 PMC2844581

[25] Kapadia-Kundu N, Dyalchand A (2006) The Pachod Paisa Scale: A Numeric Response Scale for the Health and Social Sciences. Available: http://www.ihmp.org/pp_scale_demography_india_dec19_2006.pdf. Accessed 07 July 2012.

