# Supplementary material for: User Perceptions of Shared Sanitation among Rural Households in Indonesia and Bangladesh
Source: PLoS One. 2014 Aug 4;9(8):e103886. doi: 10.1371/journal.pone.0103886 (PMC4121202; doi:10.1371/journal.pone.0103886)
Supplement: Table S1 — Research questions. (DOCX) [file pone.0103886.s001.docx]

**Table S1: Research questions**

| Research Question | Relevant survey questions included in questionnaire? | |
| --- | --- | --- |
|  | East Java | Bangladesh |
| 1. Do households with different sanitation facilities (open defecation, unimproved facility, shared improved facility, or private improved facility) experience different levels of satisfaction with their place of defecation? | X | X |
| 2. Does the level of perceived facility cleanliness drop as the number of households sharing a facility increases? | X |  |
| 3. Is there a difference in the level of satisfaction in users who perceive their facility to be clean compared to users who perceive their facility to be dirty? | X |  |
| 4. Does the level of satisfaction drop as the number of households sharing a facility increases? | X | X |
| 5. Are households with private improved facilities more satisfied than households with shared improved facilities? | X | X |
| 6. Are households with private improved facilities less likely to report open defecation than households with shared improved facilities? |  | X |
| 7. Among households who share an improved facility, are households who are satisfied with their facility less likely to report open defecation? |  | X |
| 8. Among households who share an improved facility, are households who are not satisfied with their facility more likely to have plans to build a latrine within a short time/the next year? | X | X |
