# Supplementary material for: User Perceptions of Shared Sanitation among Rural Households in Indonesia and Bangladesh
Source: PLoS One. 2014 Aug 4;9(8):e103886. doi: 10.1371/journal.pone.0103886 (PMC4121202; doi:10.1371/journal.pone.0103886)
Supplement: Table S2 — Female perceptions of household sanitation facilities in East Java. (DOCX) [file pone.0103886.s002.docx]

**Table S2: Female perceptions of household sanitation facilities in East Java**

| **Is the toilet facility safe during the day?** | |
| --- | --- |
| Yes | 1,891 (90.6%) |
| No | 196 (9.4%) |
| **Is the toilet facility safe during the night?** |  |
| Yes | 1,769 (85.0%) |
| No | 313 (15.0%) |
| **Is the toilet facility private enough?** |  |
| Yes | 1,829 (87.7%) |
| No | 257 (12.3%) |
| **Do females experience harassment during defecation or bathing?** | |
| Never/ rarely | 2,046 (98.2%) |
| Sometimes/ often | 38 (1.8%) |
