# Supplementary material for: User Perceptions of Shared Sanitation among Rural Households in Indonesia and Bangladesh
Source: PLoS One. 2014 Aug 4;9(8):e103886. doi: 10.1371/journal.pone.0103886 (PMC4121202; doi:10.1371/journal.pone.0103886)
Supplement: Variables S1 — Variable definitions. (DOCX) [file pone.0103886.s003.docx]

**Variable definitions**

*East Java*

1. *Place of defecation:* “Where do members of your household usually go to defecate?” (sanitation infrastructure options given)
2. *Sharing status:* “Do you share this facility or area with other households?” (yes, no)
   1. If yes, “How many households use this toilet facility or area?” *(number of households sharing facility is requested)*
3. *Type of sanitation:* using answers to *place of defecation* and *sharing status* questions, household is categorized as having access to a private improved, shared improved, or an unimproved facility, or as a household practicing open defecation
4. *Level of satisfaction:* “Overall, how satisfied are you with your main defecation facility?” (very satisfied, somewhat satisfied, less than satisfied, completely dissatisfied);
5. *Perceived cleanliness:* “How do you rate the cleanliness of the [sanitation facility, not including OD]?” (very clean, clean, dirty, very dirty)
6. *Improvement plans:* (if head of household indicates he or she would like to build a private latrine or improve current private latrine/toilet in response to question about changing current sanitation situation) “How likely is it that you will install a private latrine or toilet in the next 12 months?” (high, medium, low)

*Bangladesh*

1. *Sharing status:* (If owned singly) “Do any other households or persons use the latrine on a regular basis?” (yes, no)
   1. “If yes, how many households and persons use this latrine?” *(number of households and persons sharing facility is requested)*
2. *Type of sanitation:* household was categorized in dataset as having access to a private improved, shared improved, or an unimproved facility, or as a household practicing open defecation
3. *Level of satisfaction:* “How satisfied are you with your current place of defecation?” (satisfied, moderately satisfied, unsatisfied)
4. *Improvement plans:* “Do you have plans to improve the present latrine or build a new one within a short time?” (yes, to improve; yes, to build a new one; no)
5. *Open defecation:* “Does it ever happen that you or any member of your household needs to defecate in the open (other than in flood time and except small children)?” (yes, no)
6. *Ownership of the latrine:* (own, jointly owned, owned by others/neighbor, live on rent)
