# Supplementary material for: User Perceptions of Shared Sanitation among Rural Households in Indonesia and Bangladesh
Source: PLoS One. 2014 Aug 4;9(8):e103886. doi: 10.1371/journal.pone.0103886 (PMC4121202; doi:10.1371/journal.pone.0103886)
Supplement: Covariates S1 — Covariate definitions for East Java and Bangladesh. (DOCX) [file pone.0103886.s004.docx]

**Covariate definitions for East Java and Bangladesh**

*East Java*

Education level is defined as follows: ‘primary or lower’ includes playgroup, kindergarten, elementary school/Madrasah Ibtidaiyah, Kejar Paket A, Pesantren Islamic School, and never attended school; ‘secondary’ includes general/vocational junior high school/Madrasah Tsanawiyah, general/vocational senior high school/Madrasah Aliyah, and Kejar Paket B; and ‘tertiary or higher’ includes Academy D1/D2/D3, and University S1/S2/S3.   

Households were asked their primary drinking water source for both the rainy season and the dry season. Rainy season is October through April. Drinking water sources have been categorized as improved or unimproved sources using WHO/UNICEF Joint Monitoring Programme (JMP) for Water Supply and Sanitation definitions. Improved sources include: piped water into dwelling, piped water to yard/plot, public taps or standpipes, tubewells or boreholes, protected dug wells, protected springs, and rainwater. Unimproved sources include: unprotected springs, unprotected dug wells, carts with small tank/drum, tanker-trucks, surface water, and bottled water.

Sanitation status is defined using JMP definitions for improved and unimproved sanitation facilities (based on infrastructure only); sharing status is also given to clarify whether the facility itself is shared or private. Improved facilities include: flush toilets, piped sewer systems, septic tanks, flush/pour flush to pit latrines, ventilated improved pit latrines (VIP), pit latrines with slab, and composting toilets. Unimproved facilities include: flush/pour flush to elsewhere, pit latrines without slab, buckets, hanging toilets or hanging latrines, and no facilities or bush or field. A facility is considered private if the household does not share the facility with any other households. A facility is considered shared if the household shares the facility with one or more other households.

*Bangladesh tables*

Education level is defined as follows: ‘primary or lower’ includes no education, levels 1-5, and adult literacy; ‘secondary’ includes levels 6-12; ‘tertiary or higher’ includes levels 14-16.

‘Other’ occupation includes the following responses: ‘professional,’ ‘handicraft/sewing,’ ‘unemployed,’ ‘retired/elderly,’ ‘student,’ ‘child,’ ‘disabled,’ ‘stay abroad,’ and ‘other.’

Drinking water sources have been categorized as improved or unimproved sources using WHO/UNICEF Joint Monitoring Programme (JMP) for Water Supply and Sanitation definitions. Improved sources include: piped water into dwelling, piped water to yard/plot, public taps or standpipes, tubewells or boreholes, protected dug wells, protected springs, and rainwater. Unimproved sources include: unprotected springs, unprotected dug wells, carts with small tank/drum, tanker-trucks, surface water, and bottled water.

The four sanitation approaches used to work towards Open Defecation Free (ODF) communities included the following: *government only:* local governments operated with little or no assistance from nongovernmental organizations (NGOs); *government and NGO support:* local governments operated with some assistance from NGOs; *NGO, Community-Led Total Sanitation:* local governments operated with much assistance from NGOs whose methodology included Community-Led Total Sanitation (CLTS) practices; and finally, *NGO, no Community-Led Total Sanitation:* local governments operated with much assistance from NGOs whose methodology did not include CLTS practices. Community-Led Total Sanitation focuses on community mobilization and behavior change as a way to eliminate open defecation instead of the traditional method of infrastructure construction at the household level.

Sanitation status is defined using JMP definitions for improved and unimproved sanitation facilities (based on infrastructure only); sharing status is also given to clarify whether the facility itself is shared or private. Improved facilities include: flush toilets, piped sewer systems, septic tanks, flush/pour flush to pit latrines, ventilated improved pit latrines (VIP), pit latrines with slab, and composting toilets. Unimproved facilities include: flush/pour flush to elsewhere, pit latrines without slab, buckets, hanging toilets or hanging latrines, and no facilities or bush or field. A facility is considered private if the household does not share the facility with any other households. A facility is considered shared if the household shares the facility with one or more other households. Ownership status data were only available for users with private or shared improved facilities.
